# Bioturbation by the Fungus-Gardening Ant, *Trachymyrmex septentrionalis*

**DOI:** 10.1371/journal.pone.0158920

**Published:** 2016-07-08

**Authors:** Walter R. Tschinkel, Jon N. Seal

**Affiliations:** 1 Department of Biological Science, Florida State University, Tallahassee, Florida, United States of America; 2 Department of Biology, Center for Environment, Biodiversity and Conservation, University of Texas at Tyler, Tyler, Texas, United States of America; University of Sheffield, UNITED KINGDOM

## Abstract

Soil invertebrates such as ants are thought to be important manipulators of soils in temperate and tropical ecosystems. The fungus gardening ant, *Trachymyrmex septentrionalis*, is an important agent of biomantling, that is, of depositing soil excavated from below onto the surface, and has been suggested as an agent of bioturbation (moving soil below ground) as well. The amount of bioturbation by this ant was quantified by planting queenright colonies in sand columns consisting of 5 layers of different colored sand. The amount of each color of sand deposited on the surface was determined from April to November 2015. In November, colonies were excavated and the color and amount of sand deposited below ground (mostly as backfill in chambers) was determined. Extrapolated to one ha, *T*. *septentrionalis* deposited 800 kg of sand per annum on the surface, and an additional 200 kg (17% of the total excavated) below ground. On average, this mixes 1.3% of the sand from other layers within the top meter of soil per millennium, but this mixing is unlikely to be homogeneous, and probably occurs as "hotspots" in both horizontal and vertical space. Such mixing is discussed as a challenge to sediment dating by optically stimulated luminescence (OSL).

## Introduction

A variety of physical, chemical and biological processes contribute to the formation and development of the world's soils. Historically, geologists and soil scientists have emphasized the physical and chemical aspects of this process, but beginning with Darwin [1], there has been increasing awareness of the importance of biological processes in soil development [1–8]. Soils are home to thousands of species, including microbes and plants that facilitate weathering and move soluble materials around, and animals, from earthworms to aardvarks, whose burrowing brings soil to the surface (biomantling) or mixes different layers below ground (bioturbation) [3, 9–11]. These biological processes disrupt or enhance the physical and chemical processes of soil development.

In most temperate to tropical zones, ants are among the most abundant soil-dwelling animals, and are probably the premier turners of soil [12–16], often exceeding the effects of termites and vertebrates [17]. Their burrowing affects many soil characteristics such as water infiltration, grain size distribution and soil texture and mineral dissolution [6, 12–16, 18, 19], all of which can affect the soil's congeniality for organisms. Their effects are magnified because many ant species move their nests frequently [14, 20–22], and some have been shown to remodel their subterranean nests [10, 23–25], which increases bioturbation. The biomantling by ant species, or even entire communities has been studied several times [12–16], primarily by measuring the mounds of soil the ants dump on the surface on a per nest or per m^2^ basis. Reported rates vary greatly, depending on species and location. A table summarizing the published rates can be found in Tschinkel [9].

Earlier studies found significant biomantling activity and other soil movements by the Florida Harvester Ant (*Pogonomyrmex badius*) in a pine forest in northern Florida [9, 10]. While substantial, the amounts of soil surface movement by this species was much lower than biomantling in the fungus-gardening ant *Trachymyrmex septentrionalis* [26]. These results were surprising because *P*. *badius* has relatively large colonies (up to 10,000 workers) and voluminous subterranean nests (up to 10 L), whereas *T*. *septentrionalis* has much smaller nests, usually containing a few hundred workers whose average individual live weight is less than a milligram [26, 27]. The biomantling differences are probably the result of the much higher nest density of *T*. *septentrionalis*.

Despite their small size, *T*. *septentrionalis* colonies are extremely abundant. An average hectare of pine forest contains >1000 nests that collectively excavate >1 metric ton of soil each year [26]. Over the course of a millennium, these ants completely displace the top-most 6.3 cm of soil [26, 28]. As a result, *T*. *septentrionalis* is likely an important agent of biomantling in coastal plains forests, a region in which this species has a long evolutionary history [29, 30]. Furthermore, Seal and Tschinkel [26] also reported that these ants move their fungus chambers to deeper levels as the soil warms in the spring and summer, a behavior common in fungus-gardening ants in seasonal environments [30–31]. Because some shallow chambers of colonies excavated in midsummer contained sand, it seemed likely that *T*. *septentrionalis* is an important agent of bioturbation by mixing soil from different levels. The purpose of this study is to confirm and quantify this bioturbation by planting colonies in columns composed of layers of different colored sand and monitoring seasonal deposition of colored sand as well as the location of the sand in the soil column.

## Materials and Methods

### Study site

*Field site*: The study population of the fungus-gardening ant, *Trachymyrmex septentrionalis*, is located in the same 23 ha site as the *Pogonomyrmex badius* population of Tschinkel [9]. The latitude and longitude of the site are, respectively, 30.3587 and -84.4177, about 16 km southwest of Tallahassee, Florida, USA. This study site is located in the Apalachicola National Forest within the so-called sandhills ecotype [32]. Dubbed Ant Heaven, the soils are excessively drained sand but sloping toward a wetland and stream to the east. The water table is >5 m at the maximum depth to water, creating a droughty site suitable for several drought-resistant species of ants and plants (e.g. *Opuntia*). The forest is composed of longleaf pines (*Pinus palustris*) planted ca. 1975, whereas turkey oak (*Quercus laevis*), bluejack oak (*Quercus incana*), occasional sand pines (*Pinus clausa*) and sand live oak (*Quercus geminata*) occur in the subcanopy. The natural ground cover of wiregrass (*Aristada stricta*) was absent, replaced by several successional species of grasses, herbs and shrubs. *T*. *septentrionalis* nests were easily recognized in the spring by the semi-lunar crescent of excavated soil [33], and occurred at densities of about 1000 per ha.

The soil of most of Ant Heaven formed as ridges of sandy marine or dune deposits on marine limestone terraces. This Ortega Sand is strongly acidic, very well-drained and nutrient poor (85% of the nutrients are in the topmost 15cm, unpublished data, WRT). The eastern edge of Ant Heaven slopes to the Fisher Creek wetland with its very poorly drained Donovan Mucky Peat. On the west, the Ortega Sand grades into the extremely acidic Talquin Fine Sand, but little of this is included in Ant Heaven (http://websoilsurvey.nrcs.usda.gov/app/WebSoilSurvey.aspx).

This project was carried out under US Forest Service, Apalachicola National Forest permit number APA56302, Expiration Date: 12/31/2017. *Trachymyrmex septentrionalis* is not a protected species.

### Construction of layered soil

Sand of six different colors (orange, green, purple, yellow, blue, pink) was purchased from Sand Blast Entertainment (www.coloredsand.com). Using a post-hole digger, 1 m deep, 50 cm diameter holes were excavated and the excavated sand mixed with each color so that the final mixture consisted of about 25% colored sand and 75% native sand. Five different colors of these sand mixtures were layered and packed in 20 cm thick layers ("host" layers) in the excavated holes with a 10 cm layer of native sand on the top. Before layering, the sides of each hole were lined with sheer polyester fabric to prevent the ants from excavating outside the layer cake. In three of the ten "layer cakes", yellow was used instead of purple. Ten such 5-color layer cakes were constructed and allowed 2–3 weeks to settle. The order of layers and their approximate depth was native (0–10 cm), orange (10–30 cm), green (30–50 cm), purple or yellow (50–70 cm), blue (70–90 cm), pink (90–110 cm). In general, the setup of this experiment was similar to that in [23].

### Excavation and planting of colonies

In mid-April 2015, during the early stages of their annual cycle, active *T*. *septentrionalis* colonies were carefully excavated, and all ants and fungus were collected. Colonies were all broodless and contained 500 to 1000 workers. Only queenright colonies were used for the experiment.

A 30 by 30 cm, screen-bottom, metal-sided cage with a central hole in the screen bottom [23] was placed over each "layer cake". The inside walls of the cage were coated with Fluon to prevent escape. A short "starter hole" was made in the sand through the hole in the screen. A queenright colony was released in each cage. All colonies began excavating almost immediately, and had moved the workers and fungus into the growing nest within 24 hours. Evidence of their deepening nest could be seen in the colors of sand the workers brought to the surface.

**Fig 1:**
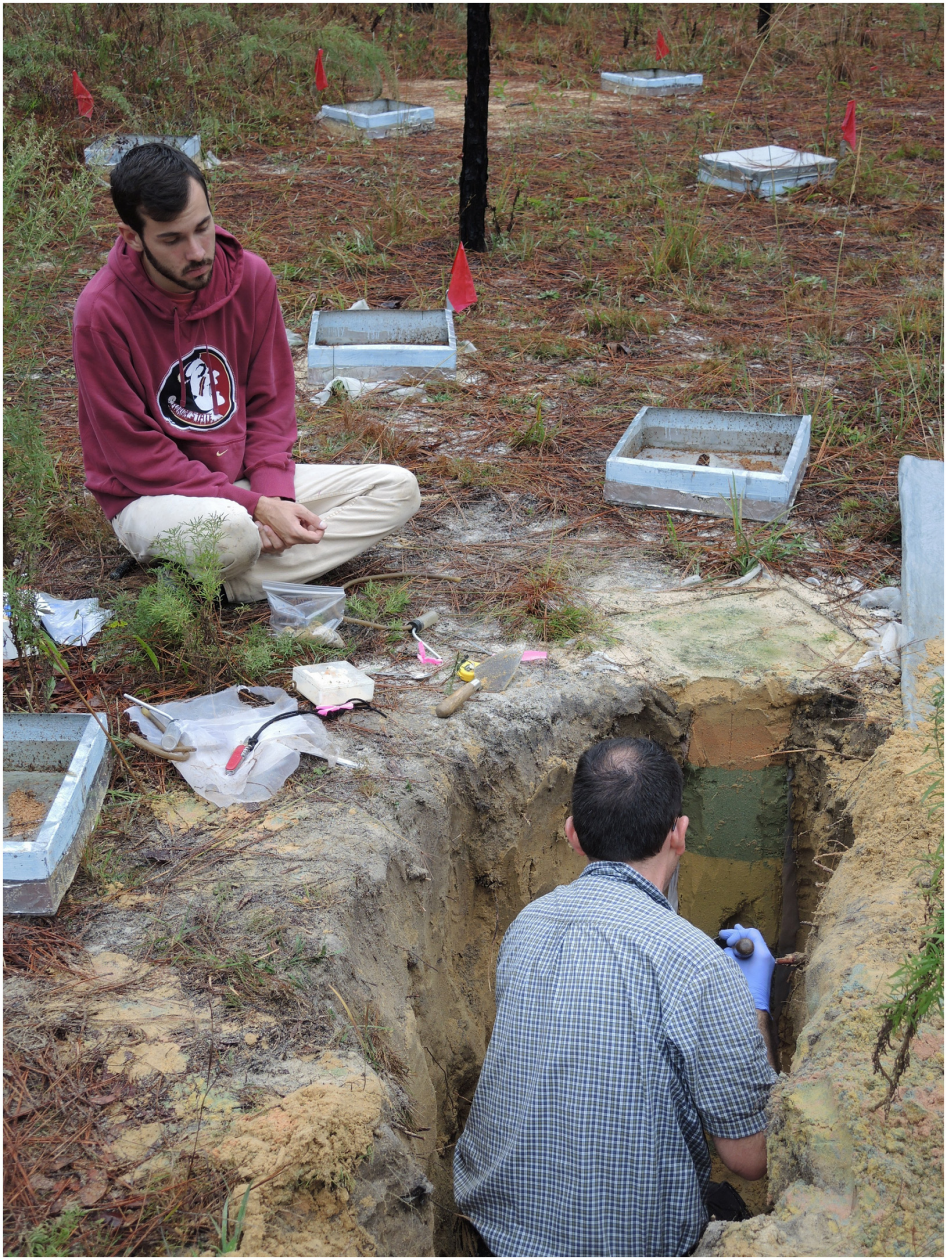
Excavation of a *Trachymyrmex septentrionalis* in progress, showing other screen-bottom cages with planted colonies. Colonies were planted in the cages in April 2015 and excavated in November 2015, at which time nine of the ten colonies survived. Written consent to use the images of these individuals was obtained.

Because the colonies were captive and had restricted foraging areas, all were located within about 30 m of one another (Fig 1) and provided with substrate for their fungus gardens twice a week. Substrates included tender young leaves from the vicinity, clusters of small flowers, wheat bran, polenta, oatmeal and cornmeal.

Ten colonies were planted, but one died early in the experiment, leaving a total of nine.

### Estimation of excavation and deposition

As the ants excavated their nests, they deposited the excavated soil on the surface in their characteristic semi-lunar crescents [33]. This sand was collected twice weekly, dried and weighed. Small samples were then photographed with a digital microscope and the number of grains of each color of sand, as well as native grains, was counted. These collections and feeding continued from April 17 until November 19–21, 2015, at which time, each colony was carefully excavated, locating each chamber. All the contents of each chamber, be they ants, fungus or backfilled sand, were collected. All collections of backfill were dried and weighed for further analysis.

Each host layer and each backfill collection was completely homogenized before each of five small samples (13–21 mg; mean 5.5 mg) was withdrawn and photographed under the digital microscope. The total number of each color and native grains was counted in these images. Total number of grains ranged from about 200 to 500. The average bulk density of these sands was about 1.5 g/cm^3^. The weight of each backfill sample could then be converted to the equivalent weight of each color-native mixture by dividing the sample weight by the proportion of colored grains in each host. Because collection of backfill from chambers inevitably also contained host layer sand, the weight of host-native sand mixture was subtracted from the computed weight of backfill. These backfill estimates therefore represent sand moved from one layer and deposited in another layer below ground. The excess weight over this computed weight was assigned to native sand.

### Data analysis

Data were compiled, analyzed and plotted in Statistica 12, using multiple regression, non-parametric tests and analysis of variance (ANOVA). Relevant data can be found in supplementary files S1, S2 and S3 Datasets.

## Results

### Above-Ground Observations

The weight and color of sand the ants deposited on the surface between April and mid-November, 2015 indicated that the colonies were both alive and actively excavating. Excavation rate was high initially (mid-April to mid-May), then, as the ants established their first fungus-filled chambers, declined to low rates or zero until late June. Thereafter deposition on the surface was small and sporadic until it increased again in September-October to decline again in November. The amount of sand deposited on the surface in the fall was much smaller than that in the spring. Deposition of colored sand varied greatly among colonies and across seasons. The quantity and timing of excavation were similar to non-caged colonies in the surrounding area and to other reports of digging behavior [26, 28].

By mid-November, each colony had deposited a mean of 758 g (s.d. 172 g) of sand on the surface (range 461–998 g). Of this, 82% (625 g; s.d. 196 g; range 269–934 g) came from colored hosts.

The color of the sand deposited on the surface before late May revealed that chambers totaling between 200 and 650 ml in volume were being excavated mostly between 30 and 70 cm below ground (7 of 9 colonies). Two of these nine colonies excavated their largest chamber between 30 and 50 cm, four between 50 and 70 cm, and one on the 50 cm boundary between colors. In two colonies, the largest chambers were shallower (10 and 30 cm). Five of the nine colonies brought up small amounts of sand from below 70 cm. Much of the sand excavated to form deeper chambers later in the summer was deposited below ground in higher chambers, as described below.

### Below-Ground Observations

**Fig 2:**
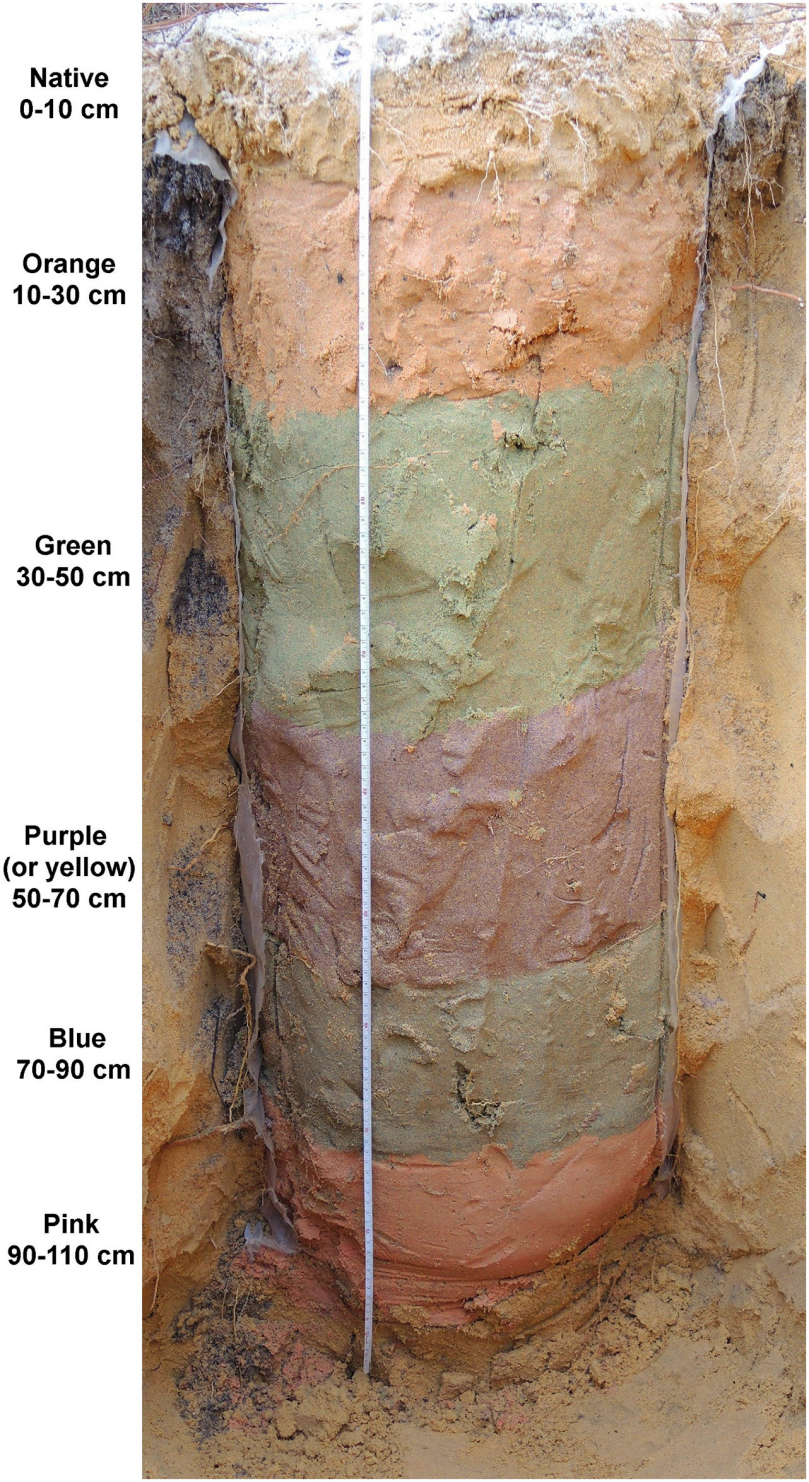
Side view of an excavated layer cake, showing the layers of colored sand, and their depth below the ground surface. The curtain of sheer fabric that surrounded the column of colored sand has been cut away, but is visible at the edges. The ants were confined to excavating their nest within this column.

Nine colonies had survived by the time of excavation, November 19–21, 2015. By this date, most colonies had finished their seasonal cycles, had ceased excavating and were preparing for winter. All nine colonies were successfully excavated to reveal the colored layers (layers and their depths shown in Fig 2) as well as backfilled or empty chambers, and any chambers that contained fungus gardens.

**Fig 3:**
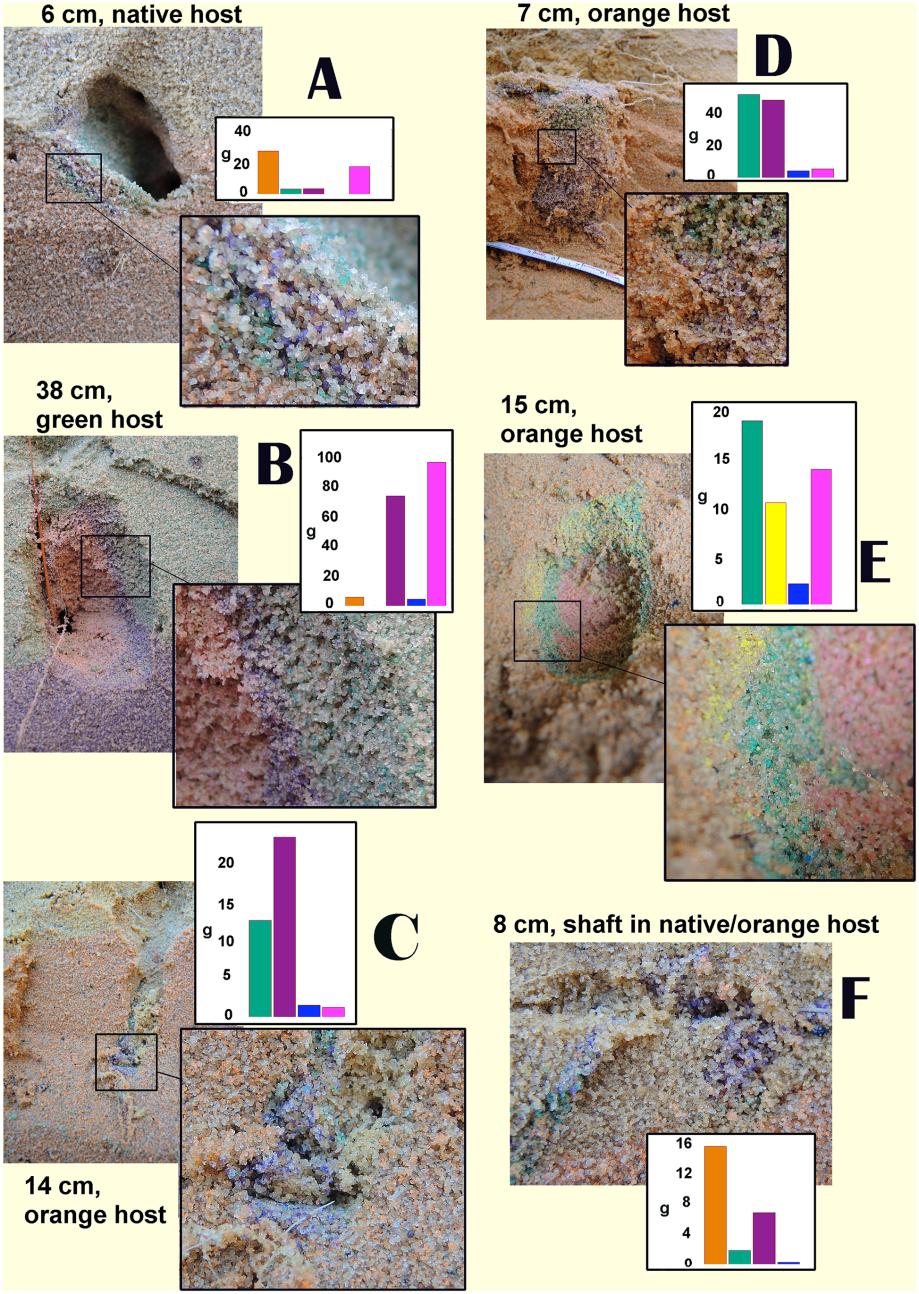
**A-F.** Examples of chambers and shafts backfilled with colored sand from other layers. Each example shows the chamber (or shaft) in general view, along with a detail at higher magnification showing the multiple colors of the backfilled sand, and a histogram showing the weight of each of the colors of backfill. These weights include the native sand contained in the mixture, but exclude the weight of the backfill originating in the host layer in which the backfill is located.

Colonies contained three to six chambers ranging in size from very small (3–6 cm^3^) to about 400 cm^3^, and ranging in depth from about 5 cm to over 100 cm. Many of these chambers had been partially or completely backfilled with sand from other layers. Examples of backfilled chambers are shown in Fig 3A–3F. Insets show details of the backfilled chamber, and clearly reveal that these consist of sand from several different source layers. The bar graphs show the weight of sand (minus host sand) from each source colored layer. Deposition ranged from linings on the floor of chambers (Fig 3A) to complete backfilling (i.e. depositing soil excavated elsewhere in existing chambers) (Fig 3B–3E). Shafts were often backfilled as well (Fig 3F). Sand was often deposited as single colors in distinct layers, suggesting that excavation in each source layer dominated for some period of time before shifting to another layer (Fig 3A, 3B and 3E). In Fig 3D, the chamber was filled with different colors from the bottom up.

The nine colonies moved a total of 1380 g of sand from one colored layer to another, with individual colonies moving from 46 g to about 330g (mean = 153 g; s.d. = 110 g). As the mean weight of soil excavated per colony was 911 g (758 g on the surface+153 g below ground), about 17% of the total excavated sand was deposited below ground.

**Fig 4:**
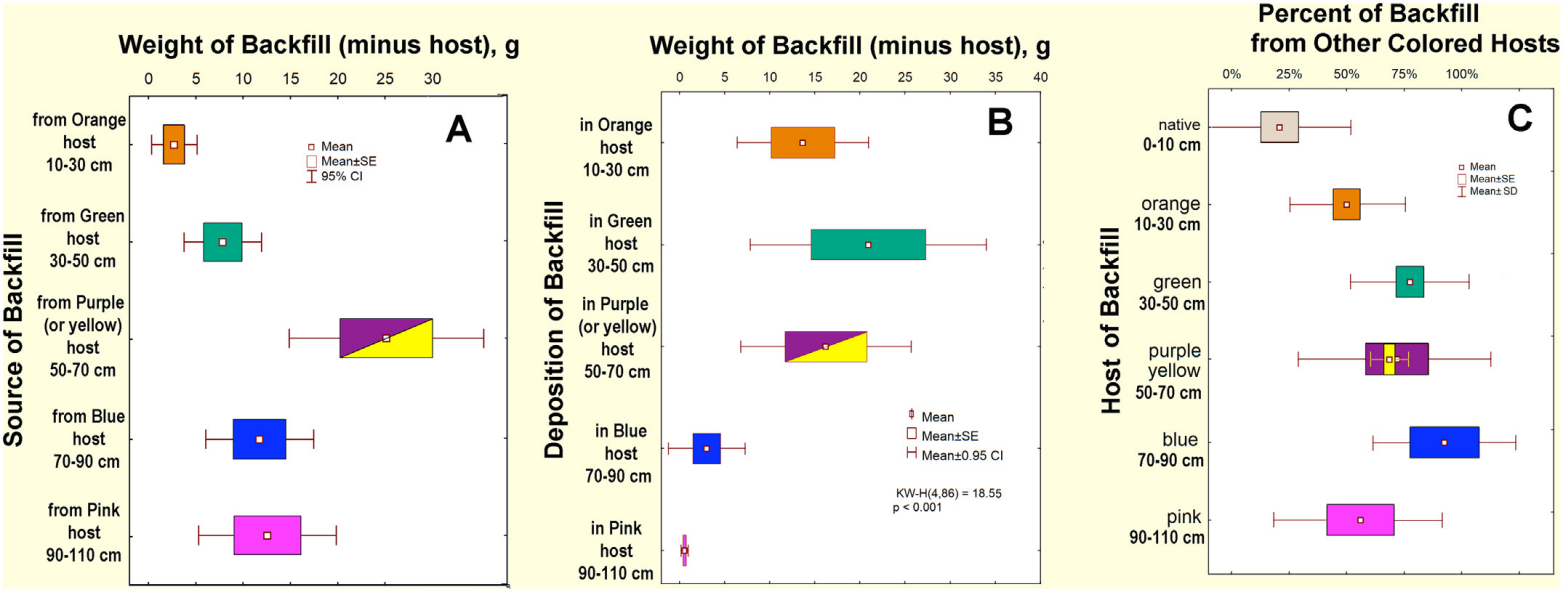
A. The mean weight (n = 9) by host source of sand deposited as backfill in chambers (excluding the weight of the host sand); B. The mean weight of backfill from other layers deposited in each host layer; C. The percent of the backfill in each host layer that came from another colored layer (n = 9). Kruskal-Wallace test by host layer (n = 5, or n = 6) showed B and C, but not A to vary significantly.

The color of the deposited sand (i.e. the host source) differed, with more sand tending to come from deeper than the green layer (30–50 cm), but this trend was not quite statistically significant (Fig 4A; purple and yellow were summed, as yellow was used instead of purple in three colonies). On the other hand, the amount of backfill deposited above the blue layer (70–90 cm) was greater (Fig 4B; Kruskal-Wallace test: KW_4,86_ = 18.55p< 0.001), suggesting that movement was primarily upward. The proportion of the backfill that came from other colored hosts was higher for the deeper layers (Fig 4C; Kruskal-Wallace test: H_6,43_ = 22.2; p< 0.001), with hosts at mid-depths receiving a higher proportion of sand from other hosts. This is the complement to the higher proportion of native backfill in the higher layers.

**Fig 5:**
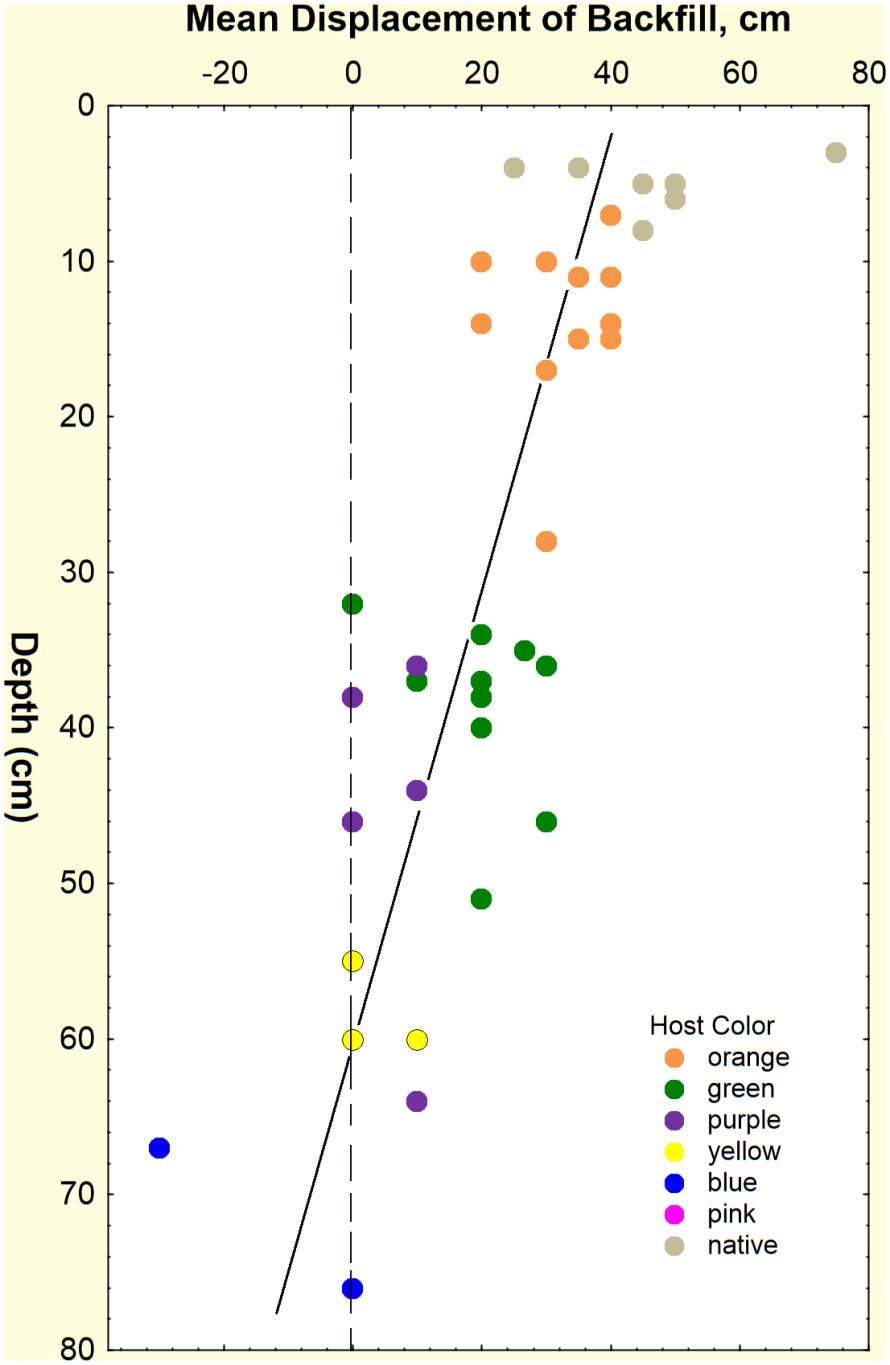
Displacement distance (cm) of the average backfilled grain of sand in relation to the depth at which it was deposited. The host color in which the grain was deposited is shown as the color of the symbols. Displacement was calculated from the mean depth of each host layer and the depth-source of each color of backfill. Most of the backfill was displaced upward, and displacement was greater for backfill originating from deeper host layers.

The distance over which an average backfill grain was displaced was estimated from the depth of each backfilled chamber, the mean depth of each source host layer and the weight of backfill derived from each of these layers. The mean distance of displacement of the backfill was highest in the upper layers (in which the backfill came from deeper) and decreased significantly with depth (Fig 5; displacement = 44–0.71*depth; F_1,38_ = 60.3; p < 0.000001), which also suggests that most displacement was upward.

**Fig 6:**
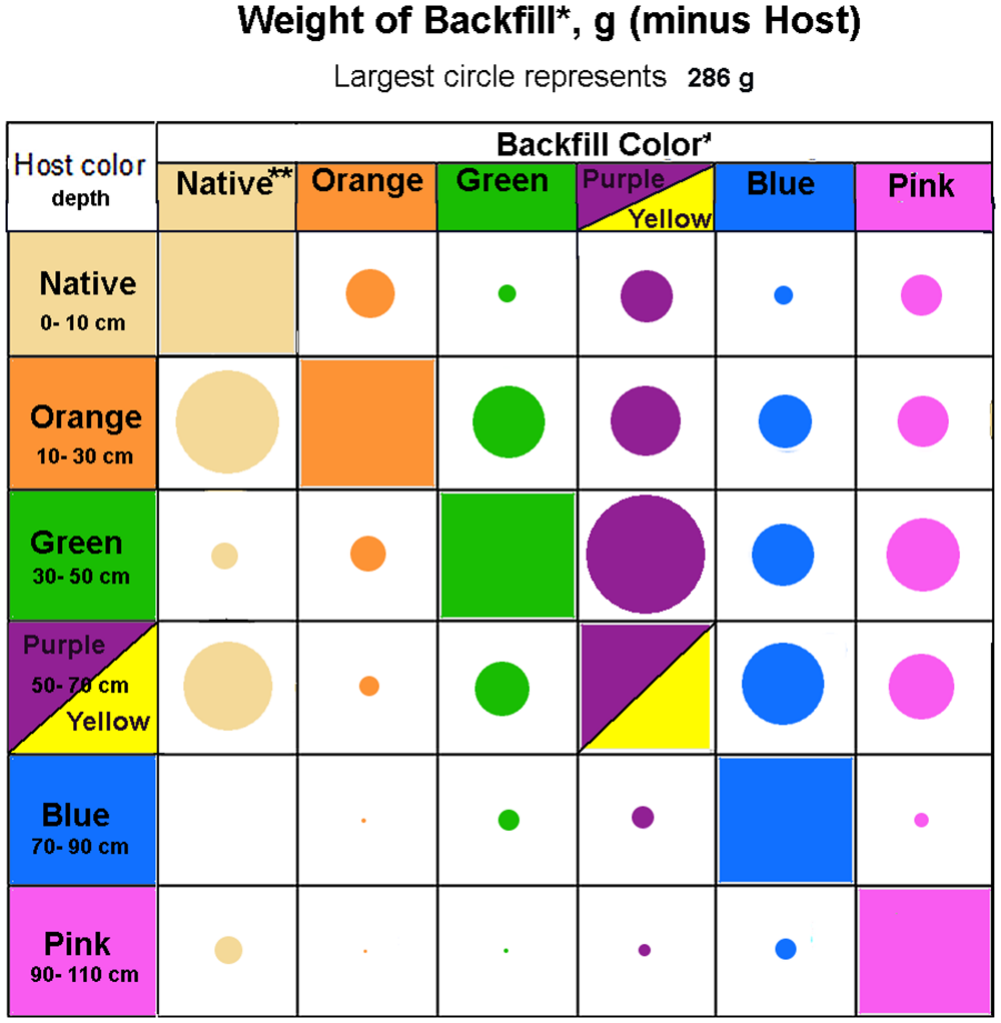
Summary of the weight of backfill deposited in each host layer, and its layer of origin. The area of the circles is proportional to the weight of sand, with the largest circle representing 286 g. Backfill originating within the same host layer as deposition was excluded from these calculations. Upward displacement greatly exceeded downward displacement, with greatest deposition tending to be in the next higher layer. "Native" was the sand weight of deposits that could not be accounted for as the diluent in the colored mixtures. Purple and yellow were combined because they occupied the same depth.

The weight of backfill from other host layers deposited in each of the different host colors quantified the movement and showed it was predominately upward (Fig 6). All colors of host received more deposits from below than above, with the highest rates being from one or two layers below for five of the six colors. Overall, the ants moved 11 times as much sand upward as downward.

The weight of sand deposited on the surface between April and November averaged 758 g per colony (s.d. 172; n = 9). Of this, 624 g (s.d. 196 g) or 82% came from colored hosts. Converted to volumes by dividing by the bulk density of sand (1.5 g/cm^3^), this indicated a mean of 505 cm^3^ of chambers (s.d. 115) had resulted from the removal of this sand, of which 416 cm^3^ were in colored hosts (s.d. 108 cm^3^). In addition, colonies also moved a mean of 146 g (s.d. = 88 g) of colored sand from one layer and deposited it below-ground in another layer. Because this sand was also excavated to form a chamber elsewhere, converting these weights to volumes yields 97 cm^3^ per colony (s.d. = 59 cm^3^). Combining the sand recovered on the surface with that deposited below ground indicates that the average colony excavated a total of 513 cm^3^ of chamber volume. The observed volume of chambers derived from direct measurements of chamber dimensions during excavation averaged 286 cm^3^ (s.d. = 154 cm^3^). A large fraction (44%) of the volume implied by the excavated sand (surface plus below-ground) is thus not accounted for. Possible reasons for this are discussed below.

## Discussion

We have previously shown the fungus-gardening ant, *T*. *septentrionalis*, to be a major agent of biomantling, that is, of moving soil from depth to the surface [26]. Our experiments now show that it is also a major agent of bioturbation that leaves almost a fifth of the soil it excavates from chambers below ground. The reason for this backfilling of existing chambers is probably due to the construction during the spring and summer of deeper fungus garden chambers, which are more conducive to the temperature and humidity sensitivity of their fungal symbiont [26, 34–37]. Presumably, the backfilling takes place during the excavation of these deeper chambers because it offers a savings in time and energy during soil transport.

The scale of this bioturbation is considerable. Seal and Tschinkel [26] found that in its typical pinelands habitat, there are over 1000 nests per ha, collectively moving over 1000 kg of soil to the surface every year, and burying the surface by over 6 cm per millennium. Our results confirm an annual deposition of about 800 kg per ha on the surface, and suggest that an additional approximately 200 kg per ha are translocated annually as backfill below ground. Because this backfill comes from several different depths, the long-term stirring of the soil strata is significant. Extrapolated to a millennium, 200 metric tons of sand, mostly within the top meter of soil, are mixed below ground by the backfilling of chambers. Averaged over a hectare to one meter depth, approximately 1.3% of the soil will have originated from deeper layers. Even so, this deposition is not uniform, being greater at middle depths (30–60 cm). Ants occasionally also make nests deeper than a meter, but this is probably a small proportion of the total deposited below ground.

A reviewer expressed concern about over-extrapolation because our sample consisted of only nine nests. In response, it is important to note that our estimates of soil movement by *T*. *septentrionalis*, both on an individual and aggregate basis, agree well with those of Seal and Tschinkel [26] whose estimates were based on about 200 nests in a range of different habitats. It thus seems likely that our per-area extrapolations are reasonable. Whether the millennial extrapolation is also reasonable is less certain, not to mention difficult to verify. This long-range extrapolation is best seen as simply an easier way to visualize a rate.

Our results are similar to those of Tschinkel [10] and Rink et al. [23] for the harvester ant, *Pogonomyrmex badius*. *P*. *badius* makes much deeper nests than *T*. *septentrionalis*, but like *T*. *septentrionalis*, it deposits a substantial portion of excavated sand (about 2.5%) below ground by backfilling or lining chambers and shafts. This amounts to about 110 g for an average sized nest (3000 cm^3^). Even though the nests of *P*. *badius* are far larger than those of *T*. *septentrionalis*, the much higher proportion of the sand deposited below ground by the latter (17%) assures that the total weight of below-ground deposits is also larger (mean, 153 g). Combining the much higher density of *T*. *septentrionalis* nests (often >1000 per ha) with their larger subsurface deposits suggests that *T*. *septentrionalis* has a larger effect on soil mixing per ha than does *P*. *badius*. Of course, both contribute to the mixing of soil, with *P*. *badius* injecting soil from much deeper into the upper 30 cm of the soil column. Estimating their relative effects is thus difficult.

Our estimates of below-ground deposition are probably conservative because they do not include movement within a host color, so that movements less than 20 cm were not included. This is probably not much of an issue because seasonal nest depths are rather uniform among seasons. For example, in summer (July and August), fungus chambers are usually deeper than 60 cm, whereas chambers in the spring and fall are typically 20–40 cm deep [26]. Thus movements within a 20 cm belt are likely very rare and minor. A puzzling outcome is that more native sand than could be explained as the diluent of the colored hosts was deposited in most layers. This could be an error introduced by our methods, or it could be that the ants actually move surface (or near-surface) sand downward in substantial amounts, or that they moved sand upward from deeper than our colored strata. The question could have been resolved if we had used colored sand as the top layer, but then, hindsight is wonderful, is it not?

The mixing of soil strata through bioturbation, creates challenges for archeologists and earth scientists who wish to apply optically stimulated luminescence (OSL) dating [38]. This method is based on the fact that when quartz sand grains are exposed to radiation (cosmic rays, radioactive elements, etc), they accumulate the capacity to emit UV light in proportion to the dose of radiation they have received [38]. This dose varies with site, and can be calibrated, but within a site, it increases with the length of exposure, which in turn is positively related to depth and time of burial. When animals bring soil to the surface, sunlight immediately zeros the luminescence, starting the accumulation of luminescence capacity over again as the soil is buried by successive layers of biomantled material. However, when soil is moved from one layer to another without exposure to light, the "alien" grains compromise the dates returned by OSL [39–42]. Deeper grains deposited among shallower ones return dates that are too old, a problem that can be addressed by taking the minimum dates derived from small samples. When shallower grains are deposited deeper, these cause an underestimation of age.

From the point of view of OSL, it is therefore important to estimate the amount of movement of grains from one level to another, as we have done in this paper. It is likely that this deposition is not spatially uniform, with greater effects at middle depths (30–60 cm), but it is also possible that the deposited soil is highly heterogeneous in horizontal space as well. Given that a *T*. *septentrionalis* nest occupies (and modifies) a soil column less than 50 cm diameter (surface area, ca. 0.2 m^2^), and assuming that the average colony moves every year, the total area affected in a millennium will be only 200 m^2^, or 2% of a hectare. This means that all of the below-ground deposits will be concentrated in only 2% of the hectare with very much larger effects on OSL dates should these hotspots happen to be sampled. Of course, other burrowing animals can blur these patterns laterally, but such information is currently lacking.

Below-ground deposition of soil has now been demonstrated for two species of ants in the field [23, 10] and one in the laboratory [24], and it seems likely that many ant species do so. Tschinkel described the nest architecture of several species of ants whose nests range from small [43–45] to large [43, 46]. The biomantling and bioturbation impacts of *T*. *septentrionalis* probably rival if not exceed the amounts of soil moved by the closely related leaf-cutting ants (*Atta* and *Acromyrmex*) [26, 47]. It is thought that the ants move most of this soil because of the relatively narrow tolerance [34–37] of their fungal symbiont toward warm temperatures and aridity. The ants move their garden deeper in the soil as the upper regions become too warm and/or dry during summer. Similar to leaf-cutting ants, the movements of soils may influence soil fertility. Attine fungus gardens are essentially a subterranean bioreactor that consumes plant fibers collected by the ants from above-ground sources (leaves, flowers, insect frass) that are either metabolized by the fungus or placed in nutrient-rich refuse depots [48–50], which depending on species, can be deposited on the surface or deep in the ground and have variable ecosystem influences [51–52]. Not only are *T*. *septentrionalis* ants annually moving relatively infertile soil up toward the surface, which displaces fertile topsoil, but the subsidence of top soil may be countered by the deposition of undigested plant fibers and other fungus garden residue that fertilizes nutrient poor soils and influences plant communities [53]. While both above and below ground fungal refuse depots have been observed at this study site in this species (JN Seal, unpublished data), only two of the nine excavated nests contained subterranean refuse deposits, which suggests that most fungal waste was distributed on the soil surface. Future work should model the potentially competing effects of movements of soil and fungal refuse on soil chemistry and microbial communities, among other mechanisms that could influence soil fertility.

The non-uniformity of some non-native soil deposits in this experiment (e.g., Fig 3B and 3E) where soils from many depths are deposited in the same chamber, is potentially an outcome of the behavioral mechanisms involved in digging. Excavation and transport of soil in most ant species is probably similarly organized as a sequence of tasks carried out by a sequence of workers in a series-parallel manner. Pellets formed at the excavation-face by one group of workers are placed nearby to be transported by other workers who only rarely carry the soil pellet all the way to the surface [54]. Such sequential transport is probably the most common source of subsurface deposition, as some of the pellets may not be picked up again, and become incorporated into the floor or walls of chambers and shafts [10]. In addition, both *T*. *septentrionalis* and *P*. *badius*, deposit a large amount of soil as backfilling in chambers. The frequency of backfilling in other ant species is not known, but the observations in this study demonstrate that there is much more to learn here.

## Supporting Information

S1 Dataset Basic colored sand data resulting from excavation of experimental colonies. (XLSX)

S2 Dataset Summed data from excavations of experimental colonies. (XLSX)

S3 Dataset Sand composition data. (XLSX)
